# Silvery Gray Hair Syndrome With Hemophagocytic Lymphohistiocytosis: A Case Report

**DOI:** 10.7759/cureus.55649

**Published:** 2024-03-06

**Authors:** Shiji Chalipat, Vishwanath Kulkarni, Sudhir Malwade, Priyanka Shah, Prasad Bijaspur

**Affiliations:** 1 Pediatrics, Dr. D.Y. Patil Medical College Hospital and Research Center, Dr. D.Y. Patil Vidyapeeth (Deemed to Be University), Pune, IND

**Keywords:** rab27a gene, silvery grey hair, immune deficiency, hemophagocytic lymphohistiocytosis (hlh), griscelli syndrome

## Abstract

Griscelli syndrome (GS) is a rare autosomal recessive disorder, which has been classified into three subtypes based on clinical and genetic differences. GS subtype 2 is commonly associated with hemophagocytic lymphohistiocytosis (HLH) and recurrent infections due to immunodeficiency. In this study, we describe a four-month-old boy with genetically proven GS2, with neurological and immunological manifestations. He presented with fever, refusal of feeds, drowsiness, and multiple episodes of seizures. Examination revealed hypopigmented skin, silvery gray hair, and organomegaly. The child developed features of HLH, fulfilling clinical and laboratory criteria. Neuroimaging findings were in concordance with HLH of the central nervous system. Microscopic examination of the hair showed clumps of melanin pigment along the hair shaft. All findings were in favor of GS type 2, complicated with HLH, which was later confirmed with a homozygous deletion of the RAB27A gene on exome sequencing. Unfortunately, the baby succumbed to death due to severe sepsis and multiorgan dysfunction. The silvery gray hair, with typical hair microscopic findings, and association with HLH are strong indicators for this potentially fatal condition and aid in prompt diagnosis and initiation of treatment. Hematopoietic stem cell transplantation is the only lifesaving treatment option.

## Introduction

Griscelli syndrome (GS) is a rare autosomal recessive immunodeficiency syndrome due to defective intravesicular trafficking mechanisms of melanosomes in melanocytes. This was first described by Griscelli and Siccardi in 1978 [1] and is characterized by deficient pigmentation of the skin and hair, and large clumps of pigment in the hair shafts. GS is classified into three types based on the genetic loci involved. GS Type 1 presents as hypopigmentation with neurological manifestations without immunodeficiency. GS Type 2 presents as hypopigmentation with immunological abnormalities, with or without neurological manifestations, and GS Type 3 presents only as hypopigmentation, without immunologic or neurological manifestations [2]. GS Type 2 is mostly diagnosed between four months to seven years of age [3], though the youngest reported is one month, with no sex predilection. It is due to RAB27A gene mutation and is associated with impaired lymphocyte and natural killer cell function. It can be associated with uncontrolled activation and proliferation of T lymphocytes and macrophages in response to infections. Activated immune cells infiltrate various organs, leading to hemophagocytosis and subsequently to a fatal condition known as hemophagocytic lymphohistiocytosis (HLH) [4]. To date, approximately 160 cases of GS have been reported in the literature, the majority from the Mediterranean and Turkish regions. Around 14 cases of GS 2 have been reported from India [4,5]. Here, we describe a four-month-old boy with genetically proven GS2, with neurological and immunological manifestations.

## Case presentation

**Figure 1:**
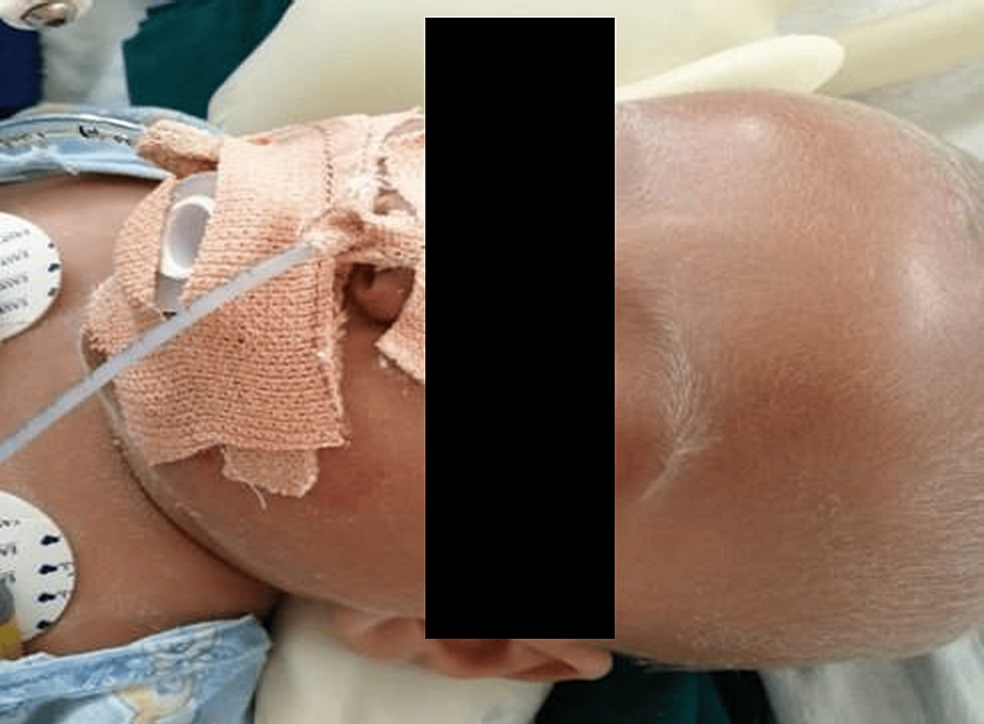
Clinical image showing a patient with fair skin, silvery gray colored hair and eyebrows

A four-month-old boy, the firstborn out of a third-degree consanguineous marriage, with an uneventful perinatal history and development until presentation, was admitted with fever, refusal of feeds, drowsiness, and multiple episodes of seizures. Examination showed no obvious dysmorphic features. The child's anthropometric parameters were appropriate for age. Table 1 depicts the hematological investigation done on day 1 of admission. He had fair skin with a silver-gray sheen on his scalp hair, eyebrows, and eyelashes. The anterior fontanelle was not bulging (Figure 1). Neurological examination revealed a depressed sensorium with a Glasgow Coma Scale (GCS) of 7/15, global hypotonia, exaggerated deep tendon reflexes, and an extensor plantar response, for which the child was shifted to the pediatric intensive care unit. Hepatosplenomegaly was present, and fundus examination revealed no abnormality.

**Table 1:** Hematological investigations on day 1 of admission

Parameter	Result	Normal Range
Hemoglobin	9.7 g/dL	11.0–14.5 g/dL
White blood cells	7900/µL	4000–12,000/µL
Neutrophils	50%	–
Lymphocytes	45%	–
Platelet count	220,000/µL	150,000–410,000 /µL
Blood sugar	133 mg/dL	70–140 mg/dL
C-reactive protein	2 mg/L	<3 mg/L
Aspartate aminotransferase (AST)	44 U/L	8–33 U/L
Alanine aminotransferase (ALT)	52 U/L	13–45 U/L
Ammonia	159 µg/dL	<50 µg/dL
Lactate	20 mmol/L	0.5–2.2 mmol/L

CSF study demonstrated no pleocytosis with an elevated protein level (62 mg/dL) and normal sugar. Because of the poor sensorium and recurrent apneic episodes, the baby required mechanical ventilation. IV fluids, inotropes, and empirical antibiotics were started. Cofactor supplementation, keeping the possibility of inborn errors of metabolism in mind, was also initiated. Electroencephalogram revealed diffuse background slowing suggestive of encephalopathy. Metabolic screening by acylcarnitine profile and urine gas chromatography showed no abnormalities. CSF viral and bacterial panel was negative. In view of these findings, whole exome sequencing was organized. The encephalopathy of the baby persisted despite seizure management. On day seven of admission, repeat hematological investigations (Table 2) revealed pancytopenia, elevated hepatic transaminases, hypertriglyceridemia, hypofibrinogenemia, hypoproteinemia, and elevated D-dimers, fulfilling the criteria of HLH. Hair microscopic examination showed clumps of melanin pigment along the hair shaft.

**Table 2:** Hematological investigations on day 7 of admission

Parameter	Result	Normal Range
Hemoglobin	5.2 g/dL	11.0–14.5 g/dL
White blood cells	3100/µL	4000–12,000/µL
Neutrophils	62%	–
Lymphocytes	30%	–
Platelets	56,000/µL	150,000–410,000/µL
C-reactive protein	160 mg/L	<3 mg/L
Aspartate aminotransferase (AST)	327 U/L	8–33 U/L
Alanine aminotransferase (ALT)	192 U/L	13–45 U/L
Triglycerides	167 mg/dL	<75 mg/dL
Fibrinogen	112 mg/dL	200–400 mg/dL
D-Dimer	1400 ng/mL	90–870 ng/mL
Serum protein (total protein)	4 g/dL	6.4–8.3 g/dL
Serum protein (albumin)	2 g/dL	3.5–5 g/dL
Serum protein (globulin)	2 g/dL	2.5–3 g/dL

**Figure 2:**
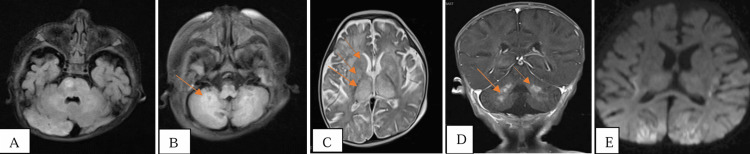
MRI brain – plain and contrast images A and B: Fluid-attenuated inversion recovery (FLAIR) axial section showing brainstem and cerebellar peduncle hyperintensities; C: T2 axial section showing basal ganglia and thalamic hyperintensities and subcortical white matter hyperintensity; D: T1 contrast axial section showing nodular contrast enhancement in cerebellar white matter; E: Diffusion-weighted image showing restricted diffusion in the bilateral occipital and insular cortex.

MRI brain, plain and contrast, was suggestive of white matter hyperintensities involving the brainstem, deep nuclei, subcortical white matter, and cerebellar peduncles, with diffusion restriction in the bilateral occipital lobe and bilateral thalamo-capsular region. The contrast MRI study showed nodular and ring-enhancing lesions (Figure 2).

The patient required escalation of inotropes and antibiotics, antifungal treatment, and intravenous immunoglobulin (IVIg). Whole blood and fresh frozen plasma were transfused for severe anemia and a deranged coagulation profile. The child was started on the induction phase of the HLH treatment 2004 protocol with dexamethasone, etoposide, and cyclosporin. Hematopoietic stem cell transplantation (HSCT) was planned, and blood samples were sent for whole exome sequencing. Unfortunately, the baby succumbed to death due to severe sepsis and multi-organ dysfunction. The whole exome sequencing report later confirmed the clinical diagnosis of GS type 2 with a homozygous deletion of the RAB27A gene on exons 3-5, classified as likely pathogenic. Genetic counseling of the parents was undertaken for Sanger sequencing and prenatal testing during the next pregnancy.

## Discussion

Managing GS requires a delicate balance between addressing the immunodeficiency and neurological components. The prognosis is variable and mostly depends on the specific subtype of GS, emphasizing the need for case-specific treatment and management plans. The distinctive feature of all three types is the partial albinism of the skin and silvery gray hair [2]. The key differences between the three types of GS are depicted below (Table 3).

**Table 3:** Classification of Griscelli's syndrome

	GS Type 1	GS Type 2	GS Type 3
Genetic locus	MYO5A gene on Chr 15q21.2 mutation	RAB27A gene Chr 15q21.3 mutation	MLPH gene on Chr 2q37 mutation
Clinical features	Hypopigmentation, neurological manifestation, no immunodeficiency	Hypopigmentation, immunological abnormalities with or without neurological manifestations	Hypopigmentation, no immunologic or neurological abnormalities
Treatment	No specific treatment	Immunochemotherapy, hematopoietic stem cell transplantation	Not required
Prognosis	Irreversible neurological disorder with long-term neurodevelopmental disability	Lethal (hemophagocytic syndrome and infection)	Good

The constellation of symptoms of silvery gray hair, with features of sepsis and HLH, raised the possibilities of GS, Chédiak-Higashi, and Hermansky-Pudlak syndrome. The presence of neurological symptoms, absence of ecchymosis, and typical hair microscopic findings ruled out Hermansky-Pudlak syndrome. Similarly, the absence of inclusions in granulocytes on peripheral blood examination and the absence of typical hair microscopic findings ruled out Chédiak-Higashi syndrome as well [6]. The classic hair microscopic finding of large melanin clumps gave the diagnostic clue toward GS. Neurological symptoms are more common in GS1 than in GS2. In GS2, neurological manifestation is not due to primary brain involvement but is actually related to the development of HLH. This is because the RAB27A gene, causative of GS2, is not expressed in neuronal cells [2]. The RAB27A gene is needed for the anchorage of melanosomes in melanocytes as well as for the exocytosis of cytolytic granules in cytotoxic lymphocytes and NK cells [7, 8]. Mutation of this gene leads to cytotoxic defects which trigger uncontrolled lymphocyte and macrophage activation, eventually leading to HLH in the accelerated phase, triggered by viral and bacterial infections leading to severe complications [4]. Usually, central nervous system (CNS) manifestations precede systemic manifestations. Therefore, the development of HLH in this baby hinted more towards GS2 than GS1.

On CSF analysis in children with CNS HLH, CSF proteinosis is one of the most common abnormalities as seen in our case [9]. The neuroimaging findings in CNS HLH are variable and can range from normal to atrophy to diffuse parenchymal lesions. The various described patterns of CNS HLH are multifocal and bilateral white matter lesions with a high rate of symmetric involvement, hyperintensities involving deep nuclei, brain stem, and cerebellum with or without necrosis, and nodular or discoid contrast enhancement. The MRI brain findings in our patient are consistent with these described patterns suggestive of CNS HLH [10, 11]. Overall, patients with GS have a complicated clinical course with recurrent infections and HLH. Their quality of life depends on the degree of neurological involvement. HLH-directed therapy followed by HSCT is the only curative treatment for patients with GS2, which needs to be undertaken promptly [12]. Without treatment, the condition is fatal. In our patient, we planned HSCT but it could not be performed as the child succumbed to death secondary to severe sepsis. Parents have been counseled for prenatal testing as we can prevent the recurrence of this autosomal recessive condition.

## Conclusions

GS2 is a rare immune deficiency disorder characterized by silvery gray hair associated with immune deficiency and is an important cause of primary HLH. The microscopic examination of the hair shaft is a simple diagnostic tool. The clinical clues, along with molecular markers of HLH, CSF, and neuroimaging findings, should prompt quick diagnosis and initiation of treatment. Hematopoietic stem cell transplantation is the only life-saving treatment option in these patients.
